# Celiac Disease and Pregnancy Outcomes in Patients with Gestational Diabetes Mellitus

**DOI:** 10.1155/2020/5295290

**Published:** 2020-10-16

**Authors:** Maria Grazia Dalfrà, Gloria Giovanna Del Vescovo, Silvia Burlina, Ilaria Baldan, Silvia Pastrolin, Annunziata Lapolla

**Affiliations:** Department of Medicine DIMED, University of Padova, Padova, Italy

## Abstract

**Aim:**

Gestational diabetes mellitus (GDM) and celiac disease, if not diagnosed and properly treated, are associated with adverse outcomes of pregnancy. The aim of our study was to examine pregnancies complicated by GDM in celiac and nonceliac women in terms of their metabolic parameters and maternal and fetal outcomes.

**Methods:**

The study involved 60 women with GDM, 20 with and 40 without celiac disease. Maternal clinical and metabolic parameters (glucose and insulin levels in the oral glucose tolerance test (OGTT), fasting plasma glucose, HbA1c, lipid profile, prepregnancy BMI, gestational weight gain, and chronic diseases), pregnancy outcomes (gestational hypertension, pre-eclampsia, eclampsia, time, and mode of delivery), and fetal parameters (weight and length at birth, and neonatal complications) were recorded.

**Results:**

The two groups did not differ significantly in maternal parameters other than blood glucose levels at 120′ in the diagnostic OGTT (141.2 ± 35.2 vs 161.2 ± 35.4, mg/dl, *p*=0.047), prepartum cLDL (127.2 ± 43.5 vs 179.6 ± 31.7 mg/dl, *p* ≤ 0.001), and total cholesterol (229.0 ± 45.9 vs 292.5 ± 42.1 mg/dl, *p* ≤ 0.001), which were significantly lower in celiac women than in nonceliac controls. Children born from celiac women had a significantly higher birth weight (3458.1 ± 409.8 vs 3209.0 ± 432.7 g, *p*=0.044) and ponderal index (2.89 ± 0.32 vs 2.66 ± 0.25 g/cm^3^, *p*=0.006) and were more likely to be large for gestational age (27.8% vs 2.5%, *p*=0.012). Analyzing the composition of the celiac and nonceliac women's diet showed that, for the same amount of kilocalories, the gluten-free diet was associated with a slight increase in the amount of carbohydrates (49.75% vs 48.54%) and a reduction in the amount of protein (21.10% vs 23.31%) and especially of fiber (9.84% vs 12.71%).

**Conclusions:**

Celiac women with GDM have much the same pregnancy outcomes as nonceliac women with GDM, except for fetal overgrowth. Gluten-free food, being richer in carbohydrates and less rich in fiber and protein, could have a role in fetal growth in celiac women.

## 1. Introduction

Gestational diabetes mellitus (GDM) is defined as diabetes diagnosed during pregnancy that was not clearly overt diabetes prior to gestation (preexisting type 2 or, very rarely, type 1 diabetes mellitus) [1]. GDM is the most common metabolic complication of pregnancy, affecting up to 14% of all pregnancies [2]. Its prevalence depends on the diagnostic criteria used and on the ethnic group considered [3, 4]. The incidence of GDM is increasing globally due to the increasing prevalence of obesity, sedentary lifestyles, and advancing maternal age. Adoption of the new, stricter diagnostic criteria proposed by the International Association of the Diabetes and Pregnancy Study Groups (IADPSG) has also contributed to its rising incidence [3, 4]. Untreated GDM is associated with adverse outcomes for both mother and fetus during pregnancy and childbirth, including pre-eclampsia, cesarean delivery, birth trauma, macrosomia, and neonatal hypoglycemia and hyperbilirubinemia [5]. GDM is also associated with severe long-term consequences, as women who develop GDM are at high subsequent risk of developing type 2 diabetes mellitus (DMT2), metabolic syndrome, and cardiovascular disease later in life [5, 6]. As for the child, intrauterine exposure to maternal hyperglycemia is associated with a higher risk of developing impaired fasting glucose (IFG), impaired glucose tolerance (IGT), DMT2, and obesity early in life [5, 6]. GDM is a complex and heterogeneous disease. In most cases, its pathophysiology is similar to that of insulin-resistance-mediated DMT2. In some patients, however, positivity for pancreatic autoantibodies (GADA Abs, ICA Abs, and IA2A Abs) may be identified during pregnancy or after delivery, making their GDM resemble type 1 diabetes mellitus (DMT1) or latent autoimmune diabetes in adults (LADA), as reported in some studies [7–9]. Depending on a pregnant woman's genetic susceptibility to diabetes, an autoimmune mechanism or insulin resistance is thought to prevail, triggering an autoimmune or nonautoimmune GDM, and a future risk of DMT1 or DMT2, respectively [9]. DMT1 is associated with other autoimmune diseases such as autoimmune thyroiditis, celiac disease (CD), uveitis, autoimmune gastritis, vitiligo, and adrenal autoimmunity [10]. It is estimated that 10–30% of diabetic patients develop other autoimmune conditions after the clinical onset of diabetes [11]. DMT1 and celiac disease have a high propensity to coexist: the prevalence of CD in DMT1 patients is 5–7 times higher than in the general population [10, 12].

CD is an autoimmune enteropathy induced by the ingestion of gluten in genetically predisposed subjects [13], and characterized by a great variety of signs and symptoms [14]. Untreated CD in pregnant women increases the risk of recurrent abortions, preterm birth, intrauterine growth restriction, and newborn with a low birth weight [15–17]. The pathogenic mechanisms underlying the reproductive disorders and obstetric complications observed in CD are not yet fully known, but nutritional deficiencies due to malabsorption and autoimmune processes seem to have a key role [16]. Inflammatory damage to the mucosa of the small intestine is responsible for a reduced absorption of iron, zinc, selenium, and folic acid, which are essential for the synthesis of gonadotropins and proper embryo development [16, 18–20]. The transglutaminases expressed in endometrial cells and placental trophoblast cells [21] are also a target for antitransglutaminase autoantibodies (anti-tTG), which can interact with cell function and negatively affect pregnancy outcomes through an immunological mechanism mediated by antigen-antibody binding [20–24]. Clinical studies on the association between CD and obstetric complications have shown that a gluten-free diet is important in reducing the risk of adverse outcomes, making celiac women's pregnancies comparable with those of women without CD [17, 20, 25, 26].

To our knowledge, the current literature lacks information on the effects of GDM in women with CD. Hence, the present study examines pregnancies complicated by GDM in women with and without CD in terms of their metabolic parameters and maternal and fetal outcomes.

## 2. Materials and Methods

The study involved 60 pregnant women diagnosed with GDM, 20 celiac, and 40 nonceliac, from among the patients treated at the ULSS 6 Diabetology Unit in Padova between January 2011 and March 2019. GDM was diagnosed with a 75 g oral glucose tolerance test (OGTT) according to the IADPSG criteria [27] and national guidelines [28]. Celiac women were enrolled retrospectively by searching among patients treated at our diabetes clinics who presented with a medical code for a diagnosis of CD in their medical records. The nonceliac women with GDM were randomly chosen from among our patients with GDM, matched for prepregnancy BMI, age, gestational week at diagnosis of GDM, and year of delivery.

The study was conducted in accordance with the Declaration of Helsinki and its later amendments, and was approved by the local ethics committee.

At first visit, all the women's anthropometric clinical data were recorded, including age, prepregnancy body weight and BMI, height, chronic diseases, family history, and obstetric history. The women were followed up by a multidisciplinary team comprising a gynecologist, a diabetologist, a dietician, and a nurse specialized in diabetes and pregnancy.

All the women with GDM were given a standard diet consistent with the nutritional needs of the mother and fetus, and aiming to ensure an adequate maternal metabolic control and weight gain in accordance with the guidelines of the Institute of Medicine (IOM) [29] and the American Diabetes Association (ADA) [30]. The diet given to celiac patients included only gluten-free foods. All women were trained to monitor their own glucose levels at home and were asked to record fasting and one-hour postprandial blood glucose levels, and food intake in a diary. Patients attended follow-up visits every 2–4 weeks based on individual needs. Insulin treatment was started when fasting plasma glucose was >90 mg/dl and/or 1 h postprandial plasma glucose was >130 mg/dl. During the routine visits, patients' weight, blood pressure, glycemic levels, lipid profile and HbA1c, and fetal growth data obtained from ultrasonography were recorded.

Eight to 12 weeks after delivery, a 75 g OGTT was performed in 14 celiac and 39 nonceliac women and the results led to patients being reclassified as having a normal glucose tolerance (NGT) or an altered glucose tolerance (AGT). AGT includes the following conditions: impaired fasting glucose (IFG), impaired glucose tolerance (IGT), and DMT2. The women's lipid levels and pregnancy outcome data were also recorded.

The maternal outcomes recorded were as follows: gestational hypertension, pre-eclampsia, eclampsia, time, and mode of delivery. As neonatal outcomes, we considered weight and length at birth, shoulder dystocia, hypoglycemia, hyperbilirubinemia, neonatal asphyxia, and congenital malformations. The newborns were classified as large for gestational age (LGA) if their birth weight was > 90^th^ percentile, and small for gestational age (SGA) if it was <10^th^ percentile, according to standard growth and development tables for the Italian population [31]. Macrosomia was diagnosed for a birth weight of more than 4000 g. The ponderal index (PI) was calculated using the Rohrer formula: PI = weight (g)/height^3^ (cm^3^). HbA1c was measured using standard high-performance liquid chromatography [32].

## 3. Statistical Analysis

Continuous variables are expressed as means ± standard deviations, and distributions were assessed using Student's *t*-test and the Kruskall–Wallis test for independent samples. Categorical variables are presented as proportions, and the two groups were compared for categorical data using the *χ*^2^ test. A 95% confidence interval was considered for all tests, and a *p* < 0.05 was deemed statistically significant. The IBM SPSS 25 was used for the statistical analyses.

## 4. Results

Clinical and metabolic parameters are shown in Table 1

The two groups did not differ significantly in mean maternal age, prepregnancy BMI, maternal morbidity, weight gain during pregnancy, gestational age of GDM diagnosis, HbA1c at diagnosis, or need for insulin treatment. Autoimmune thyroid diseases (Hashimoto thyroiditis and Graves disease) were more frequent in the celiac group (15% vs 2.5%), though the difference was not statistically significant (*p*=0.067), probably due to the small sample size. The composition of the celiac and nonceliac women's diet was analyzed using the information drawn from the nutritional panels concerning the patients' food intake. This analysis showed that, for the same amount of kilocalories (range 2000–2200 Kcal), the gluten-free diet was associated with a slightly higher intake of carbohydrates (49.75% vs 48.54%), and a lower amount of protein (21.10% vs 23.31%) and especially of fiber (9.84% vs 12.71%) (Table 2).

As concerning maternal pregnancy outcomes, there was only one case of gestational hypertension in the control group. The mode of delivery was a cesarean section for 22.2% of the CD group vs 33.3% in the control group (*p*=0.853). CD patients had significantly lower blood glucose levels than controls at 120′ (141.2 ± 35.2 vs 161.2 ± 35.4 mg/dl, *p*=0.047) of the diagnostic OGTT. As for lipid profiles, the celiac women had significantly lower values than controls for prepartum cLDL (127.2 ± 43.5 vs 179.6 ± 31.7 mg/dl, *p* ≤ 0.001) and total cholesterol (229.0 ± 45.9 vs 292.5 ± 42.1 mg/dl, *p* ≤ 0.001).

**Figure 1:**
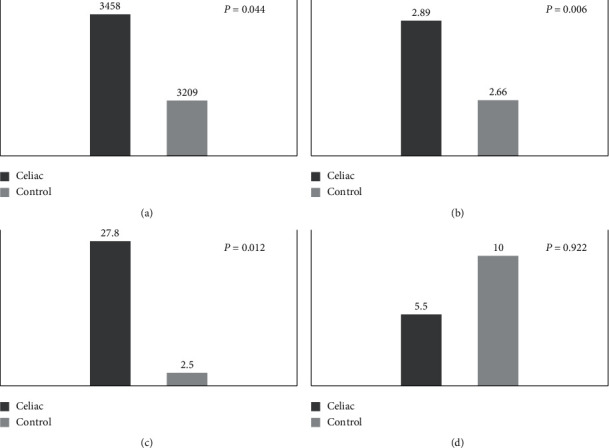
(a) Birth weight (grams). (b) Ponderal index (g/cm^3^). (c) LGA (%). (d) SGA (%).

The children born from celiac women had significantly higher values for birth weight (3458.1 ± 409.8 vs 3209.0 ± 432.7 g, *p*=0.044), PI (2.89 ± 0.32 vs 2.66 ± 0.25 g/cm^3^, *p*=0.006), and frequency of LGA (27.8% vs 2.5%, *p*=0.012). Macrosomia only occurred in the CD group, affecting 11.1% of the newborn, and the frequency of SGA was lower among the celiac mothers than in the control group (5.5% vs 10%, *p*=0.922) (Figure 1). No neonatal complications were recorded in either group.

After delivery, the group of celiac women had a higher frequency of AGT (21.4% vs 15.4%, *p*=0.825), and—at the follow-up OGTT—they had higher glucose levels at 0′ (93.6 ± 6.7 vs 87.8 ± 7.8, *p*=0.016) and lower glucose levels after 30 minutes (122.6 ± 21.6 vs 151.8 ± 20.1 mg/dl, *p*=0.004) than the nonceliac control group. Lipid profiles after delivery did not differ between the two groups (data not shown).

## 5. Discussion

Our study confirms that the outcome of pregnancies complicated by GDM in treated women with CD is much the same as in women without CD who develop GDM. These findings are in agreement with the literature, which shows that a known diagnosis of CD and strict adherence to a gluten-free diet are fundamental to reducing the risk of obstetric complications [17, 20, 25, 26].

Our study also demonstrates for the first time an accelerated fetal growth in celiac pregnancies complicated by GDM. In fact, the most important finding of the present study was that children born to celiac mothers had a significantly higher birth weight, PI, and likelihood of LGA—unlike the increased frequency of SGA newborn among undiagnosed celiac women reported in the literature [15–17].

We hypothesize that the differences in fetal growth parameters identified in our sample could be explained by the different composition of the two groups' diets. Diet has a key role in the treatment of both GDM and CD, and managing the two conditions together is complicated by the fact that gluten-free options often have a higher glycemic index and contain larger quantities of carbohydrates and fats, as reported in several studies [10, 33–35]. Careful analysis of the diet prescribed to our celiac women (based on the nutritional panels on the gluten-free foodstuffs they consumed) showed that, for the same intake of kilocalories, using gluten-free foods and gluten-free-rendered products changed the composition of their macronutrients intake compared with the diet of the nonceliac controls. Specifically, there was a slight increase in the intake of carbohydrates and a reduction in the quantity of fiber, which might be responsible for the accelerated fetal growth in the celiac group.

Moreover, our celiac mothers had lower prepartum lipid levels than the control group, probably due to an altered intestinal absorption. Data on the lipid profile of celiac women in pregnancy are lacking in the literature, but studies that compared the lipid profiles of the celiac population with healthy [36, 37] and diabetic [38] populations found lower levels of total cholesterol, LDL, and triglycerides in celiac groups, even those on gluten-free diets. Even if they are not directly comparable, our results are therefore consistent with other published reports.

Finally, our celiac group had lower blood glucose levels at 60′ and 120′ of the OGTT during pregnancy, and at 30′, 60′, and 120′ of the early follow-up OGTT after delivery. These results are in agreement with older [39, 40] and more recent [41, 42] studies documenting a lower glucose absorption in people with CD. On the other hand, the celiac group's glycemia was higher at 0′ of both the diagnostic and the follow-up OGTTs than in women with GDM alone. This finding does not disagree with those reported by other authors [42] and is compatible with the condition of celiac disease, in fact fasting glycemia does not depend on the intestinal absorption capacity but reflects the hepatic insulin sensitivity [43, 44].

The main strengths of this study lie in that this was the first to investigate the effects of GDM in women with CD and that it documented for the first time a higher frequency of LGA babies born to women with GDM and CD. Although our findings are limited by the small sample size and the retrospective nature of our research, they confirm the important role of dietary intervention in the treatment of GDM and suggest that more attention should be paid to the composition of a gluten-free diet for pregnant women with CD.

## 6. Conclusions

In conclusion, the two groups considered in this study showed differences in their glucose and lipid profiles, probably associated with the differences between gluten containing and gluten-free diet and with the altered intestinal absorption in celiac women. Our study demonstrates that celiac mothers who develop GDM have pregnancy outcomes no different from nonceliac women with GDM, except for fetal overgrowth. This would suggest that gluten-free food, being richer in carbohydrates and less rich in fiber and protein, could influence intrauterine fetal growth.

These findings increase our knowledge on the impact of celiac disease on the outcomes of GDM patients and their babies, and can serve as a basis for future studies in larger samples.

## Figures and Tables

**Table 1:** Clinical and metabolic characteristics of 20 women with GDM and CD and 40 women with GDM without CD (data are means ± standard deviations).

		GDM celiac(*n* = 20)	GDM control (*n* = 40)	*p* value
Age (yrs)		35.4 ± 4.1	35.1 ± 4.2	0.791
Prepregnancy BMI (kg/m^2^)		22.3 ± 4.6	22.3 ± 4.5	0.997
Weight gain (kg)		10.7 ± 4.2	10.5 ± 4.5	0.824
Maternal morbidity (%)		35% (7/20)	15% (6/40)	0.853
Autoimmune thyroiditis (%)		15% (3/20)	2.5% (1/40)	0.067
Gestational week of GDM diagnosis		22.9 ± 4.3	24.0 ± 4.0	0.354
HbA1c at diagnosis (%)		5.12 ± 0.26	5.07 ± 0.36	0.667
Diagnostic OGTT (mg/dl)	Plasma glucose 0′	88.3 ± 7.8	85.6 ± 8.9	0.261
	Plasma glucose 60′	159.6 ± 40.2	176.7 ± 34.7	0.105
	Plasma glucose 120′	141.2 ± 35.2	161.2 ± 35.4	0.047
Insulin therapy (%)		10% (2/20)	20% (8/40)	0.620
Prepartum lipid profile (mg/dl)	Total cholesterol	229.0 ± 45.9	292.5 ± 42.1	≤0.001
	HDL	67.9 ± 12.3	70.9 ± 21.7	0.634
	LDL	127.2 ± 43.5	179.6 ± 31.7	≤0.001
	Triglycerides	169.8 ± 98.6	212.2 ± 76.5	0.161
Gestational week of delivery		39.0 ± 1.1	38.9 ± 1.2	0.884
Follow-up OGTT (mg/dl)	Plasma glucose 0′	93.6 ± 6.7	87.8 ± 7.8	0.016
	Plasma glucose 30′	122.6 ± 21.6	151.8 ± 20.1	0.004
	Plasma glucose 60′	127.5 ± 45.2	141.3 ± 28.4	0.320
	Plasma glucose 120′	98.4 ± 23.4	97.4 ± 27.0	0.908
AGT		21.4% (3/14)	15.4% (6/39)	0.825

**Table 2:** Composition of macronutrients in the diet of women with vs without CD.

Components	Gluten-free GDM diet (%)	Standard GDM diet (%)	Difference
Protein	21.10	23.31	−2.21%
Lipids	26.72	28.15	−1.37%
Carbohydrates	49.75	48.54	+1.21%
Total fiber/1000 kcal	9.84	12.71	−2.87

## Data Availability

The data used to support the findings of this study are included within the article.
